# First report of *Theileria annulata* in Nigeria: Findings from cattle ticks in Zamfara and Sokoto States

**DOI:** 10.1186/s13071-021-04731-4

**Published:** 2021-05-07

**Authors:** Adamu Haruna Mamman, Vincenzo Lorusso, Babagana Mohammed Adam, Goni Abraham Dogo, Kevin J. Bown, Richard J. Birtles

**Affiliations:** 1 School of Science, Engineering and Environment, The University of Salford, Greater Manchester, Salford, M5 4WT UK; 2 Department of Veterinary Parasitology and Entomology, Faculty of Veterinary Medicine, University of Jos, Plateau State, Jos, Nigeria; 3 Global Research & Intellectual Property Division, Vetoquinol, Paris, France

**Keywords:** Ticks, Cattle, Tick-borne pathogens, Tick-borne diseases, *Theileria* spp., Piroplasms, Livestock, Nigeria, Africa

## Abstract

**Background:**

Ticks and tick-borne pathogens (TBPs) represent a significant economic burden to cattle farming in sub-Saharan Africa including Nigeria. However, in the northern part of this country, where the largest livestock population resides, little is known about the contemporary diversity of ticks and TBPs. This area is particularly vulnerable to climate change, undergoing marked transformation of habitat and associated flora and fauna that is also likely to include ticks. This study aimed to document the occurrence of tick species and Apicomplexan TBPs in cattle from north-western Nigeria.

**Methods:**

In 2017, ticks were collected from cattle in Zamfara and Sokoto States and identified morphologically. Additionally, a subset of ticks was screened molecularly for the detection of apicomplexan DNA.

**Results:**

A total of 494 adult ticks were collected from 80 cattle in Zamfara and 65 cattle in Sokoto State. Nine tick species were encountered, among which the presence of one, *Hyalomma turanicum*, had not previously been recorded in Nigeria. *Hyalomma rufipes* was the most prevalent tick infesting cattle in Zamfara State (76%), while *Hyalomma dromedarii* was the most prevalent in Sokoto State (44%), confirming the widespread transfer of this species from camels onto livestock and its adaptation to cattle in the region. Of 159 ticks screened, 2 out of 54 (3.7%) from Zamfara State and 29 out of 105 (27.6%) from Sokoto State harboured DNA of *Theileria annulata*, the agent of tropical theileriosis.

**Conclusions:**

This study confirms the presence of a broad diversity of tick species in cattle from north-western Nigeria, providing the first locality records for Zamfara State. The occurrence of *H. turanicum* indicates a distribution of this tick beyond northern Africa. This study provides the first report for *T. annulata* in Nigerian ticks. Given its enormous burden on livestock farming in north Africa and across Asia, further investigations are needed to better understand its epidemiology, vector transmission and potential clinical significance in cattle from northern Nigeria and neighbouring Sahelian countries.

**Graphical abstract:**

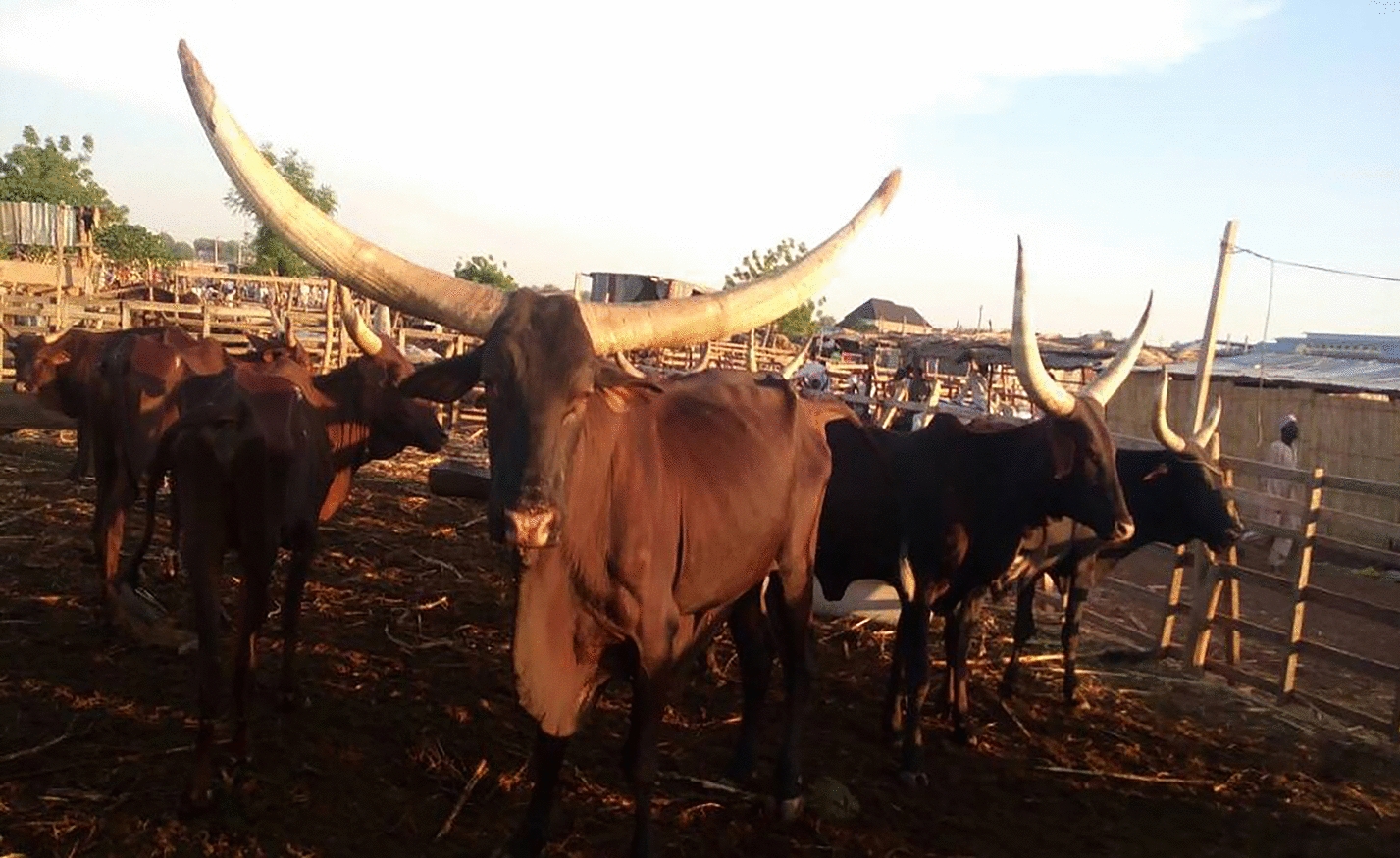

## Background

Ticks represent a significant economic burden to cattle farming and, overall, the development of the livestock sector in sub-Saharan Africa (SSA) [1–3]. Their significance is due to the impairment they cause to livestock productivity, attributable to both the direct and indirect effects of tick’s parasitism and blood feeding [2]. In cattle, direct damage caused by ticks include anaemia, stress (‘tick worry’), reduction of feeding and thus decrease of weight gain and milk yields, susceptibility to secondary infections, devaluation of hide quality, hypersensitivity and toxicosis [1, 2]. Indirect adverse consequences of tick infestation in cattle are linked to the conditions that are caused by the plethora of tick-borne pathogens (TBPs), including mostly protozoa and bacteria, but also helminths, viruses and fungi, some of which are of zoonotic importance [1–4]. The most important TBPs threatening cattle health and productivity in SSA are the causative agents of theileriosis (i.e. the ‘East Coast fever’ agent *Theileria parva*; *Theileria annulata*; *Theileria mutans* and *Theileria velifera*), babesiosis (i.e. *Babesia bigemina* and *Babesia bovis*), anaplasmosis (i.e. *Anaplasma marginale*, *Anaplasma centrale* and *Anaplasma bovis*) and ehrlichiosis (i.e. the ‘heartwater’ agent *Ehrlichia ruminantium*) [1].

Despite the enormous burden of ticks and TBPs on livestock farming, for many parts of SSA, even fundamental epidemiological information is lacking. Nigeria is a case in point; despite one of the largest cattle populations in the continent (of approximately 20 million heads) [5], contributing one third of national agricultural GDP and providing 36.5% of the total protein intake of Nigerians [6], substantial gaps affect the current understanding of the epidemiology of ticks and TBPs in the country [7], with knowledge of cattle-associated tick diversity and distribution being rather patchy [8–12] when not outdated [13–16]. Additionally, although approximately 90% of the country’s cattle population is concentrated in the northern region [6, 17], most historical surveys were carried out in southern States [13, 15]. So far, published investigations on cattle ticks from northern Nigeria have focused on limited areas of eastern (e.g. Maiduguri and Yobe State) [10, 11], or western States (e.g. Sokoto and Kebbi States) [18–21], limiting in some instances the identification of ticks to the genus level [10, 11, 19]. Similarly, the majority of studies on TBPs in Nigeria, detecting the presence of apicomplexan parasites belonging to the genera *Theileria* and *Babesia* and members of the bacterial genera *Anaplasma*, *Ehrlichia*, *Rickettsia* and *Coxiella*, have mostly relied on cytological (i.e. microscopical examination of blood smears and biopsies) [22–27] and serological approaches (e.g. ELISA and immunofluorescence assays) [25, 28–31] and only in a few, recent instances on the molecular screening of bovine blood [32–34] and ticks [35, 36].

The epidemiological importance of surveying ticks and TBPs in cattle from northern Nigeria is enhanced by the frequent movement and introduction in this region of livestock hailing from neighbouring countries like Niger, Chad and Cameroon, brought to Nigeria to be sold in more profitable local markets [37]. Furthermore, the heavy reliance on climate-sensitive economic activities, such as agriculture and livestock keeping, makes northern Nigeria particularly vulnerable to climate change [38]. Spanning the Sudano-Sahelian ecological zone [39, 40], this region is currently experiencing a combination of rising heat and declining rainfall that together are accelerating desert encroachment and marked habitat change [38, 41, 42]. Besides affecting cattle health directly through their effects on water and pasture availability, these alterations may also lead to indirect negative consequences, linked to the likely changes that they will cause on tick populations’ diversity and ecology [43]. Habitat changes may indeed compromise the fitness of some endemic tick species and create new niches exploitable by exotic species, originating from Sahelian and north African countries, adapted to hot and dry environments. The arrival of such species may well be accompanied by the TBPs they vector.

The present study aimed therefore to determine the contemporary diversity of cattle-associated ticks and apicomplexan TBPs in a region of north-western Nigeria heavily reliant on cattle keeping and significantly affected by climate change [42, 44, 45], with the objective of assessing the extent of change that may be be attributed to the latter’s impact.

## Methods

### Study area

**Fig. 1:**
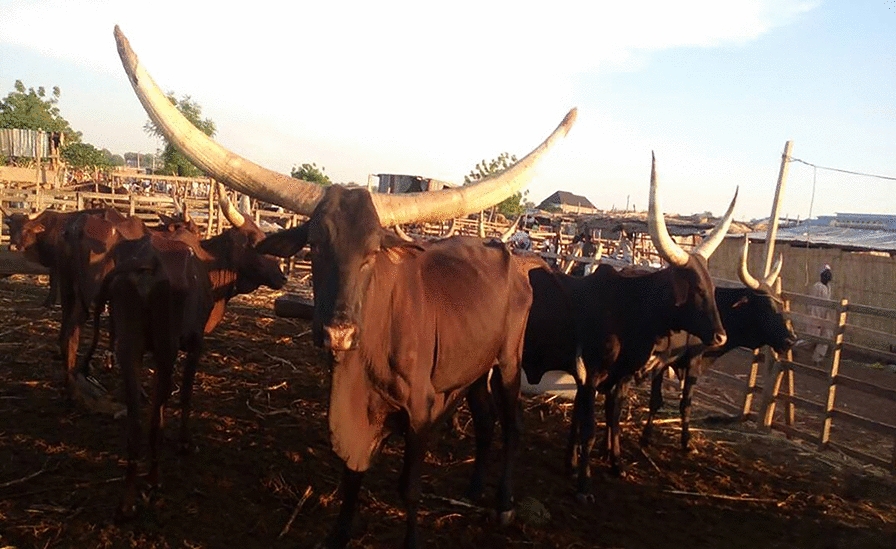
Cattle sampled at one of the market sites (i.e. Illela) in Sokoto State

Field activities were carried out between March and May 2017, in two north-western States of Nigeria, namely Zamfara and Sokoto, where a convenience sample of cattle were inspected and surveyed for tick infestations. In Zamfara, ticks were collected from cattle in the villages of Anka, Kwaye, Kwakwalwa, Gema and Abara, all of which lie within the Anka Local Government Area (LGA) (11°59′N and 6°02′E). In Sokoto, ticks were collected from cattle in cattle markets from three LGAs, namely Sokoto North, Wurno and Illela (13°03′N and 5°14′E) (Fig. 1; Table 1). Both Sokoto and Zamfara are among the poorest States of the Federal Republic of Nigeria [46]. Their economy is almost entirely reliant on agriculture. Livestock including cattle, sheep and goats are reared, and some crops are grown. Donkeys and camels are commonly used as draft animals [44, 47]. The region lies on the boundary of the Sudan savanna and Sahel climatic zones [40]. The meteorology is seasonal, with a 3–4 month wet season occurring between June and September, during which time about 500 mm of rain falls. The remainder of the year is very dry. Average daily temperatures range between about 18 °C and 38 °C. The vegetation is characterised by open savanna grasslands or open savanna woodland, with fine- and broad-leaved trees and shrubs, which are deciduous for several weeks [48].

**Table 1:** Number of animals surveyed, number of ticks collected and mean tick loads

State	Local government area (LGA)	Village	No. of cattle sampled	Ticks collected			Mean tick count/animal ± SE	
				Males	Females	Total	Village level	State level
Zamfara	Anka	Anka	16	36	6	42	2.62 ± 1.67	3.0 ± 2.28^a^
		Kwaye	14	22	14	36	2.57 ± 2.31	
		Kwakwalwa	11	28	15	43	3.9 ± 1.87	
		Gema	15	32	14	46	3.06 ± 2.12	
		Abara	24	56	17	73	3.04 ± 2.88	
Sub-total			80	174	66	240		
Sokoto	Sokoto North	Kara	13	18	36	54	4.15 ± 4.20	3.91 ± 3.15^a^
	Wurno	Achida	20	40	54	94	4.70 ± 3.87	
	Illela	Illela	32	29	77	106	3.31 ± 1.91	
Sub-total			65	87	167	254		
Total			145	261	233	494		

No statistically significant difference between mean tick burdens (P = 0.8)

### Tick collection and identification

Each animal sampled was restrained by its owner/herder and its hide examined carefully, focusing in particular on established predilection sites for tick attachment (i.e. ear, dewlap, abdomen, hooves, inguen, perineum, peri-anal region and tail) [8, 49]. From these anatomical regions, all visible adult ticks were collected using steel forceps to remove each specimen in its entirety. Immediately after collection, all ticks removed from the same individual cattle were placed in a 5-ml plastic tube containing 70% ethanol, before being transported to the University of Salford for further analysis. Once in the laboratory, all collected ticks were identified to the species level on the basis of observed anatomical features, using the taxonomical keys by Walker et al. [50].

### Detection and identification of Apicomplexa using molecular methods

A subset of collected ticks (*n* = 159, 32.2% of total ticks) were screened for apicomplexan pathogens using molecular methods. These ticks were chosen to embrace all species encountered and the different locations in which each tick species was encountered (Table 3).

Crude DNA extracts, prepared from individual ticks as previously described [51], were incorporated into a previously described PCR targeting an 18S rDNA fragment specific to apicomplexan taxon [52]. PCRs were prepared in a dedicated DNA-free laboratory. “Blanks” (PCRs containing water instead of DNA extracts) were co-processed with all samples at a ratio of 5 samples:1 blank, to test for cross-contamination. Reagent controls (a DNA-free negative and a *Babesia microti* positive) were also included in each set of PCRs prepared.

The success of the PCR was assessed by UV visualisation of GelRed-stained amplification products (of about 680 base pairs) following their electrophoretic resolution on a 1% (w/v) agarose gel. Amplification products were purified using an Isolate II PCR and gel kit according to the manufacturer's instructions (Invitrogen, Carslbad, CA, USA). Sanger sequencing of both strands of each PCR product was carried out commercially. Chromatograms obtained were visualised using Chromas Pro software (Technelysium, Brisbane, Australia). Data from complementary strands of each amplicon were aligned with one another, and regions of ambiguity together with primer sequences at the extremities were removed. The identity of the organism from which a sequence obtained was determined by comparison with data held on GenBank using Basic Local Alignment Search Tool (BLAST).

### Statistical analysis

Data were entered in Microsoft Excel, through which mean tick infestations and standard errors were calculated at the study village, LGA and State (i.e. Zamfara and Sokoto State) level (see Table 1). For the two States, mean prevalence, including 95% confidence intervals (CI), of tick species retrieved were calculated  employing the WinPepi software (version 11.6). Using the same software, cumulative tick counts recorded for each State were compared statistically by chi-squared test. In addition, infection rates were compared according to the tick species and State of provenance (for both ticks and cattle), using the two-tailed Fisher’s exact test. *P* values < 0.05 were considered statistically significant.

## Results

### Tick identification and infestation burden

**Fig. 2:**
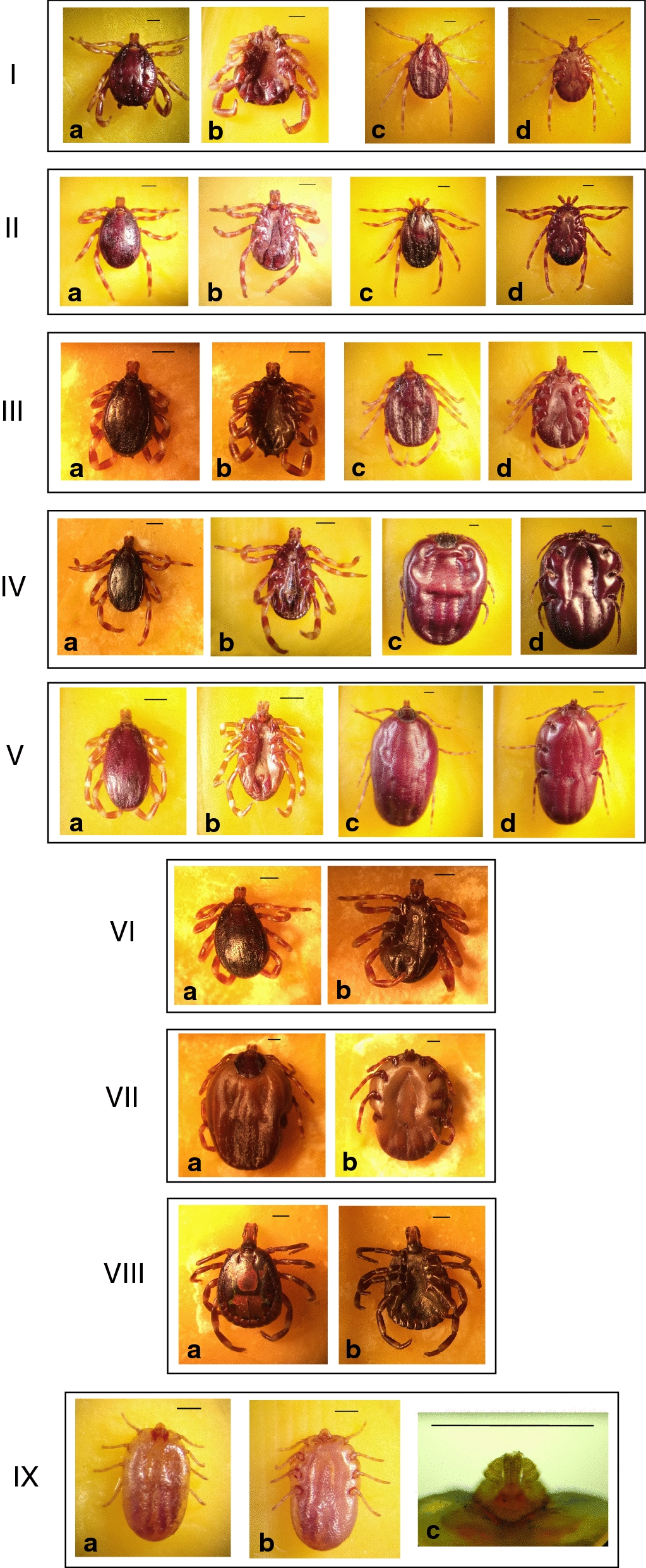
Tick species encountered in this survey. From top to bottom: *Hyalomma dromedarii* (**I**): adult male, dorsal and ventral view (**I, a–b**), and adult female, dorsal and ventral view (**I, c–d**); *Hyalomma rufipes* (**II**): adult male, dorsal and ventral view (**II, a-b**), and adult female, dorsal and ventral view (**II, c–d**); *Hyalomma impeltatum* (**III**): adult male, dorsal and ventral view (**III, a–b**), and adult female, dorsal and ventral view (**III, c–d**); *Hyalomma truncatum* (**IV**): adult male, dorsal and ventral view (**IV, a–b**) and adult female, dorsal and ventral view (**IV, c–d**); *Hyalomma impressum* (**V**): adult male, dorsal and ventral view (**V, a–b**) and adult female, dorsal and ventral view (**V, c–d**); *Hyalomma marginatum* (**VI**): adult male, dorsal and ventral view (**VI, a–b**); *Hyalomma turanicum* (**VII**): adult female, dorsal and ventral view (**VII, a–b**); *Amblyomma variegatum* (**VIII**): adult male, dorsal and ventral view (**VIII, a–b**); *Rhipicephalus (Boophilus) decoloratus* (**IX**): adult female, dorsal and ventral view (**IX, a–b**) and details of the ventral view of the mouthparts (**IX, c**) showcasing 3 + 3 rows of hypostomal teeth and the protuberance with pectinate setae on the internal margin of palp article I. Black bar = 1 mm

In total, 494 adult ticks were collected from 145 cattle: of these, 254 were off 65 cattle in Sokoto and 240 off 80 cattle in Zamfara (Table 1). The mean infestation rate was 3.0 in Zamfara and 3.9 in Sokoto, with no statistically significant difference being recorded (*P* = 0.8) (Table 1). A total of nine tick species were encountered; these included seven *Hyalomma* species (i.e. *Hyalomma dromedarii*, *Hyalomma impeltatum*, *Hyalomma impressum*, *Hyalomma marginatum*, *Hyalomma rufipes*, *Hyalomma truncatum* and *Hyalomma turanicum*), *Amblyomma variegatum* and *Rhipicephalus* (*Boophilus*) *decoloratus* (Fig. 2, Table 2). All nine species were present in Zamfara, and these included *H. turanicum* (Fig. 2VII, a–b), recorded for the first time in Nigeria. Only five species were present in Sokoto, namely (from the most to the least prevalent) *H. dromedarii*, *H. impeltatum*, *H. truncatum, H. impressum* and *H. rufipes* (Table 2). However, these corresponded to five of the six most abundant species collected in Zamfara, with only those species for which a single specimen was found (i.e. *H. marginatum*, *H. turanicum* and *Rh. (Bo.) decoloratus*) and *A. variegatum* being absent (Table 2). There was a marked difference between the relative abundance of tick species in each State. In Zamfara, *H. rufipes* dominated (76.2%), with no other species accounting for > 8% of samples. Conversely, in Sokoto, *H. dromedarii* was the most abundant (43.7%) and, together with *H. impeltatum*, accounted for 80% of the ticks collected (Table 2). *Hyalomma rufipes* was present in Sokoto, but at a relative prevalence of only 3.1%, whereas *H. dromedarii* was present in Zamfara, but only at a relative prevalence of 2.5% (Table 2). Overall, 261 male ticks and 233 female ticks were collected. The ratio of male to female ticks was very different in the two States, being 2.6:1 in Zamfara and 1:1.9 on Sokoto (Table 2).

**Table 2:** Cumulative counts, prevalence, number of males and females, and male:female ratio of ticks identified

State	Tick species	Total	Mean Prevalence % (95% confidence interval)	Males	Females	Male: female ratio
Zamfara	*Hyalomma rufipes*	183	76.2 (70.3–81.5)	135	48	2.8: 1
	*Hyalomma truncatum*	18	7.5 (4.5–11.6)	16	2	8: 1
	*Hyalomma impeltatum*	14	5.8 (3.2–9.6)	6	8	1: 1.3
	*Hyalomma impressum*	9	3.7 (1.7–7.0)	8	1	8: 1
	*Amblyomma variegatum*	7	2.9 (1.2–5.9)	7	0	7: 0
	*Hyalomma dromedarii*	6	2.5 (0.9–5.3)	1	5	1: 5
	*Hyalomma marginatum*	1	0.4 (0.0–2.3)	1	0	1: 0
	*Hyalomma turanicum*	1	0.4 (0.0–2.3)	0	1	0: 1
	*Rhipicephalus* (*Boophilus*) *decoloratus*	1	0.4 (0.0–2.3)	0	1	0: 1
Total		240		174	66	2.6: 1
Sokoto	*Hyalomma dromedarii*	111	43.7 (37.5–50.0)	47	64	1: 1.4
	*Hyalomma impeltatum*	93	36.6 (30.7–42.9)	29	64	1: 2.2
	*Hyalomma truncatum*	27	10.6 (7.1–15.1)	7	20	1: 2.9
	*Hyalomma impressum*	15	5.9 (3.3–9.5)	0	15	0: 15
	*Hyalomma rufipes*	8	3.1 (1.4–6.1)	4	4	1: 1
Total		254		87	167	1: 1.9

### Tick-borne pathogens

Out of the total of 159 ticks screened molecularly, 31 ticks (19.5%) were found positive for apicomplexan DNA, with all bar two being ticks from Sokoto (Table 3). Significantly more ticks from Sokoto (29/105 tested, 27.6%) yielded a PCR product than ticks from Zamfara (2/54, 3.7%) (*P* < 0.001) and significantly more cattle from Sokoto (18/35 tested, 51.4%) bore infected ticks than cattle from Zamfara (2/35 tested, 5.7%) (*P* < 0.001).

**Table 3:** Ticks screened and tested positive for the detection of apicomplexan (i.e. *Theileria annulata*) DNA

Tick species	Zamfara		Sokoto	
	Proportion of screened ticks/total (percentage)	Proportion of positive ticks for *Theileria annulata* (Prevalence)	Proportion of screened ticks/total (percentage)	Proportion of positive ticks for *Theileria annulate * (prevalence)
*H. rufipes*	32/183 (17%)	1/32 (3.1%)	5/8 (62.5%)	2/5 (40%)
*H. truncatum*	7/18 (38 .9%)	0/7 (0%)	6/27 (22.2%)	3/6 (50%)
*H. impeltatum*	5/14 (35.7%)	0/5 (0%)	38/93 (40.9%)	11/38 (28.9%)
*H. impressum*	2/9 (22.2%)	0/2 (0%)	7/15 (46.7%)	1/7 (14.3%)
*A. variegatum*	3/7 (42.9%)	0/3 (0%)	0	–
*H. dromedarii*	2/6 (33.3%)	1/2 (50%)	49/111 (44.14%)	12/49 (24.5%)
*H. marginatum*	1/1 (100%)	0/1 (0%)	0	–
*H. turanicum*	1/1 (100%)	0/1 (0%)	0	–
*Rh. (Bo.) decoloratus*	1/1 (100%)	0/1 (0%)	0	–
Total	54/240 (22.5%)	2/54 (3.7%)^a^	105/254 (41.3%)	29/105 (27.6%)^a^

^a^Statistically significant difference (*P* < 0.001) between infection rates

Unambiguous sequence data were obtained from 23 of the amplicons, including those obtained from the two positive ticks from Zamfara. BLAST analysis of these sequences (609 base pairs) revealed all to be indistinguishable from one another and to share 100% similarity with partial 18S rDNA sequences of many strains of *Theileria annulata* (e.g. GenBank MN944852). The 18S rDNA sequence obtained shared < 99.4% similarity with those from other *Theileria* species (most similar was 4 SNPs compared to *Theileria lestoquardi* 18S rDNA, GenBank AF081135). A selected sequence amongst those obtained was deposited in GenBank on 30 October 2020 (GenBank MW191850). Tick species containing *T. annulata* DNA included mainly *H. dromedarii* and *H. impeltatum*, but also *H*. *truncatum, H*. *impressum* and *H*. *rufipes* (i.e. all species encountered in Sokoto) (Table 3). The prevalence of infection in each tick species present in Sokoto did not vary significantly (*P* = 0.6), ranging from 14.3% in *H. impressum* to 50% in *H. truncatum* (Table 3). The large majority (*n* = 27/31; 87.1%) of PCR-positive ticks were females, assessed as being either partially (*n* = 16) or very engorged (*n* = 11) (data not shown). However, four PCR-positive ticks were male, one of which (i.e. *H. dromedarii*) appeared to be unfed.

## Discussion

The rich diversity of tick species parasitising cattle encountered in this survey is generally in keeping with contemporary reports in Nigeria [8, 9]. As expected for sites in the Sahel, and as previously reported for Sokoto, *Hyalomma* species dominated [18, 20, 50, 53–56]. However, the presence of such a high prevalence of *H. dromedarii* is remarkable (Fig. 2I, a–d). This tick has a wide range in northern Africa and beyond and is the predominant species parasitizing camels across this range [19]. Numerous previous reports demonstrate its ability to infest other hosts including cattle, but not with the relative success we observed [53, 57]. In Sokoto, *H. dromedarii* accounted for almost half the total number of collected ticks, suggesting frequent transfer of ticks from camels to cattle and/or that *H. dromedarii* has adapted itself to parasitise cattle here to a degree not reported elsewhere in its range [58]. That the dominance of *H. dromedarii* on cattle in Sokoto was not also observed in Zamfara suggests that the ecological niche it currently occupies in Sokoto does not extend southward within the Sudan savanna of Nigeria [58]. In Zamfara, *H. rufipes* was by far the most abundant tick encountered (Table 2; Fig. 2II, a–d). This species is the most widespread member of the genus present in Africa and its major contribution to cattle-associated tick fauna has been reported at several sites across this range [54, 59, 60], including in northern Nigeria [8, 9, 18, 20].

Perhaps the most noteworthy encounter among cattle-associated ticks in Zamfara was *H. turanicum* (Fig. 2VII, a–b). This species has, to our knowledge, not been reported in Nigeria or elsewhere in West Africa previously. It is thought to be endemic in the north-east of the continent and is established in arid, hot parts of southern Africa after accidental introduction [50]. This tick is not known to transmit pathogens to livestock, although it is considered a vector of the Crimean-Congo haemorrhagic fever virus to humans [50]. *Hyalomma turanicum* has a two-host life cycle, with adults typically parasitising wild and domesticated large ruminants and larvae and nymphs feeding on smaller mammals and ground-frequenting birds [50]. The tick has also been reported in Europe, associated with migratory birds using the western European-African flyway [61]. As this flyway embraces Nigeria and large parts of Africa north of the Sahara, it is reasonable to propose that the *H. turanicum* observed in Zamfara was introduced as a feeding nymph by a migratory bird. As yet it is too early to conjecture whether *H. turanicum* is established in northern Nigeria; further surveys of cattle and likely hosts of immature life-stages would help clarify this uncertainty.

Undoubtedly, the most unexpected finding of this study is the presence of *T. annulata* in north-western Nigeria. We are unaware of any previous reports of tropical theileriosis in Nigeria, or of any reports of *T. annulata* detection in ticks in the country. Tropical theileriosis is recognised as one of the most economically important diseases of livestock across north Africa and much of Asia [32], and its presence in Nigeria, where livestock productivity is already severely comprised by endemic parasites and pathogens [33], is an additional concern. Our detection of *T. annulata* DNA in ticks collected off 18 (of 35 tested) cattle in three different markets in Sokoto as well as off two (of seven tested) cattle in one village in Zamfara suggests it may be established in north-western Nigeria. That Sokoto is a likely port of introduction, be it recent or not, is not unexpected as it is a major centre for livestock (including camel) trade in the region, attracting farmers and pastoralists not just from north-western Nigeria, but also from neighbouring Niger and further afield in the Sahel and Saharan regions [26, 62]. The importance of trans-border trade as routes of entry of exotic ticks and TBPs into Nigeria has been established in the south-west of the country [63]. Further characterisation of the *T. annulata* populations detected in Sokoto using, for example, previously described polymorphic markers [64] and comparison of these data with those obtained elsewhere in the parasite’s range may help pinpoint their provenance.

Of the four *Hyalomma* species implicated in the transmission of *T. annulata* in Africa [65], only *H. dromedarii* was encountered on cattle in this study. Although our study detected *T. annulata* DNA in several *Hyalomma* species, all but one specimen were partially fed or near replete; thus, results cannot be interpreted as an indication of vector competence. The only unfed tick specimen that was found positive in this survey was a male *H. dromedarii*, further implicating this species in the transmission of *T. annulata* in the region. That this tick species, which is adapted to hot and dry habitats, is likely to thrive in north-western Nigeria as climate change provokes greater environmental aridity [65] has clear implications for the future epidemiology of *T. annulata* infections in the region.

## Conclusions

This study confirmed the presence of numerous tick species associated with cattle in north-western Nigeria. Noteworthy is the preponderance of *H. dromedarii* in cattle from Sokoto, highlighting the suitability of this tick species for the arid environments of the Sahelian belt [50]. The occurrence of *H. turanicum*, recorded for the first time in Nigeria, indicates a distribution of this tick beyond northern Africa. Perhaps of most importance, we present clear evidence for the presence of *T. annulata*, the agent of tropical theileriosis, in north-western Nigeria and demonstrate its carriage by a range of primarily fed ticks collected off cattle. These observations pave the way for further epidemiological studies to clarify the transmission of *T. annulata* infections in the region and demand veterinary investigation of their impact on livestock well-being and productivity.

## Data Availability

All data generated or analyzed during this study are included in this published article and its additional files.

## References

[1] Uilenberg G (1995). International collaborative research: significance of tick-borne haemoparasitic diseases to world animal health. Vet Parasitol.

[2] Jongejan F, Uilenberg G (2004). The Global importance of ticks. Parasitology.

[3] Minjauw B, McLeod A. Tick-borne diseases and poverty: The impact of ticks and tick-borne diseases on the livelihoods of small-scale and marginal livestock owners in India and eastern and southern Africa. Research Report, DFID Animal Health Programme. Centre for Tropical Veterinary Medicine, University of Edinburgh. 2003;p.116.

[4] Dantas-Torres F, Chomel BB, Otranto D (2012). Ticks and tick-borne diseases: A one health perspective. Trend Parasitol.

[5] FAO. Africa Sustainable Livestock 2050. Transforming livestock sector. Nigeria. What do long-term projections say? 2019. http://www.fao.org/in-action/asl2050/countries/nga/en/. Accessed 23 Oct 2020.

[6] World Bank, Livestock Productivity and Resilience Support Project (P160865) 2017. http://documents.worldbank.org/curated/en/479121500403272629/pdf/ITM00184-P160865-07-18-2017-1500403268591.pdf. Accessed 23 Oct 2020.

[7] Oguntomole O, Nwaeze U, Eremeeva M (2018). Tick-, flea-, and louse-borne diseases of public health and veterinary significance in Nigeria. Trop Med Infect Dis.

[8] Lorusso V, Picozzi K, de Bronsvoort B, Majekodunmi A, Dongkum C, Balak G (2013). Ixodid ticks of traditionally managed cattle in central Nigeria: where *Rhipicephalus (Boophilus) microplus* does not dare (yet?). Parasit Vect.

[9] Kamani J, Apanaskevich D, Gutiérrez R, Nachum-Biala Y, Baneth G, Harrus S (2017). Morphological and molecular identification of *Rhipicephalus (Boophilus) microplus* in Nigeria, West Africa: a threat to livestock health. Exp Appl Acarol.

[10] Opara MN, Ezeh NO (2011). Ixodid ticks of cattle in Borno and Yobe states of Northeastern Nigeria: breed and coat colour preference. Anim Res Int.

[11] Musa HI, Jajere SM, Adamu NB, Atsanda NN, Lawal JR, Adamu SG (2014). Prevalence of tick infestation in different breeds of cattle in Maiduguri. Northeastern Nigeria Bangl J Vet Med.

[12] Eyo JE, Ekeh FN, Ivoke N, Atama CI, Onah IE, Ezenwaji NE (2014). Survey of tick infestation of cattle at four selected grazing sites in the tropics. Glob Vet.

[13] Dipeolu OO (1975). The incidence of ticks of *Boophilus* species on cattle, sheep and goats in Nigeria. Trop Animal Health Prod.

[14] Mohammed AN (1977). The seasonal incidence of ixodid ticks of cattle in Northern Nigeria. Bull Anim Health Prod Afr.

[15] Iwuala MOE, Okpala I (1978). Studies on the ectoparasitic fauna of Nigerian livestock I: types and distribution patterns on hosts. Bull Anim Health Prod Afr.

[16] Bayer W, Maina JA (1984). Seasonal pattern of tick load in Bunaji cattle in the subhumid zone of Nigeria. Vet Parasitol.

[17] Awogbade M (1979). Fulani pastoralism and the problems of the Nigerian Veterinary Service. Afr Aff.

[18] Lawal MD, Fabiyi JP, George BDJ, Adamu Y, Kabir A, Alayande MO (2017). Preliminary study on the Monthly dynamics of cattle tick infestation in Sokoto, north western Nigeria. Nig J Anim Prod.

[19] Opara MN, Abdu Y, Okoli IC (2005). Survey of ticks of Veterinary Importance and Tick-borne Protozoa of Cattle grazed in very hot months in Sokoto Municipality. Nigeria Int J Agric Rural Dev.

[20] Okwuonu ES, Bala AY, Ikpeze OO (2017). Ticks infestation of Zebu cattle crosses in Sokoto Nigeria. Bioscientist.

[21] Abdullahi YA, Magami IM, Audu A, Mainasara MM (2018). Prevalence of ticks on camels and cattle brought to Dodoru market Kebbi state Nigeria. Path Sci.

[22] Leeflang P, Ilemobade AA (1977). Tick-borne diseases of domestic animals in northern Nigeria. I. Historical review, 1923–1969. Trop Anim Health Prod.

[23] Leeflang P, Ilemobade AA. Tick-borne diseases of domestic animals in northern Nigeria. II. Research summary (1966). to 1976. Trop Anim Health Prod.

[24] Akinboade OA, Dipeolu OO (1984). Comparison of blood smear and indirect fluorescent antibody techniques in detection of haemoparasite infections in trade cattle in Nigeria. Vet Parasitol.

[25] Saidu SN, Abdulkadir IA, Akerejola OO (1984). *Theileria mutans* infection in Nigerian cattle. Trop Anim Health Prod.

[26] Dipeolu OO, Amoo A (1984). The presence of kinetes of a *Babesia* species in the haemolymph smears of engorged *Hyalomma* ticks in Nigeria. Vet Parasitol.

[27] Kamani J, Sannusi A, Egwu O, Dogo G, Tanko T, Kemza S (2010). Prevalence and significance of haemoparasitic infections of cattle in north-central Nigeria. Vet World.

[28] Obi TU (1978). Survey of the incidence of anaplasmosis among Nigerian Zebu trade cattle. Trop Anim Health Prod.

[29] Ajayi SA, Fabi JP, Umo I (1982). Clinical bovine anaplasmosis and babesiosis in Fresian cattle: an outbreak in Nigeria and its control. Wld Anim Rev.

[30] Ajayi SA, Dipeolu OO (1986). Prevalence of Anaplasma marginale, Babesia bigemina and B bovis in Nigerian cattle using serological methods. Vet Parasitol.

[31] Elelu N, Bankole AA, Musa RJ, Odetokun IA, Rabiu M, Biobaku KT (2020). Serospatial epidemiology of zoonotic *Coxiella burnetii* in a cross section of cattle and small ruminants in northern Nigeria. PLoS ONE.

[32] Elelu N, Ferrolho J, Couto J, Domingos A, Eisler MC (2016). Molecular diagnosis of the tick-borne pathogen *Anaplasma marginale* in cattle blood samples from Nigeria using qPCR. Exp Appl Acarol.

[33] Lorusso V, Wijnveld M, Majekodunmi AO, Dongkum C, Fajinmi A, Dogo AG (2016). Tick-borne pathogens of zoonotic and veterinary importance in Nigerian cattle. Parasit Vectors.

[34] Hector E, Elelu N, Ferrolho J, Couto J, Sanches G, Antunes S, Domingos A, Eisler M (2019). PCR detection of *Ehrlichia ruminantium* and *Babesia bigemina* in cattle from Kwara State, Nigeria: unexpected absence of infection. Parasitol Res.

[35] Ogo N, de Mera I, Galindo R, Okubanjo O, Inuwa H, Agbede R (2012). Molecular identification of tick-borne pathogens in Nigerian ticks. Vet Parasitol.

[36] Lorusso V, Gruszka KA, Majekodunmi A, Igweh A, Welburn SC, Picozzi K (2013). Rickettsia africae in Amblyomma variegatum ticks Uganda and Nigeria. Emerg Infect Dis.

[37] ECOWAS - SWAC/OECD. Livestock and regional market in the Sahel and West Africa. Potentials and challenges. 2008. https://www.oecd.org/swac/publications/41848366.pdf. Accessed 30 Oct 2020.

[38] Ebele NE, Emodi NV (2016). Climate change and its impact in Nigerian economy. J Sci Res Report.

[39] FAO. Irrigation in Africa in figures–AQUASTAT Survey, (2005). Nigeria Irrigation in Africa in figures AQUASTAT Survey – 2005 Edited by Karen Frenken. FAO Water reports, 29. Rome.

[40] Fishwick RW, Kaul RN (1970). Sahel and Sudan zone of northern Nigeria, north Cameroons and the Sudan. Afforestation in Arid Zones.

[41] Abdulkadir TS, Salami AW, Aremu AS, Ayanshola AM, Oyejobi DO (2017). Assessment of neural networks performance in modelling rainfall amounts. J Res Forest Wildl Environ.

[42] Haider H. Climate change in Nigeria: Impacts and responses. K4D Helpdesk Report 675. Brighton, UK: Institute of Development Studies. 2019;1–38.

[43] Ogden NH, Lindsay LR (2016). Effects of climate and climate change on vectors and vector-borne diseases: ticks are different. Trends Parasitol.

[44] Atedhor GO (2015). Agricultural vulnerability to climate change in Sokoto State Nigeria. African J Food Agric Nutr Dev.

[45] Chukwuji NC, Aliyu GT, Sule S, Yusuf Z, Zakariya J. Awareness, access and utilization of information on climate change by farmers in Zamfara State, Nigeria. Libr Philos Pract*.* 2019;1–24.

[46] National Bureau of Statistics. Poverty and Inequality in Nigeria: Executive Summary – 2019. 2019; 1–25. National Bureau of Statistics, Plot 762, IndependenceAvenue, Central Business District, Abuja, FCT, Nigeria. https://nigerianstat.gov.ng/elibrary?queries[search]=poverty. Accessed 23 Oct 2020.

[47] Bello M, Anka A, Yusuf A. Declining Grazing Resources: the Stateand Future of The Livestock Economy in Zamfara. IOSR Journal Of Humanities And Social Science. 22: 61–72.

[48] Keay R (1949). An example of Sudan zone vegetation in Nigeria. J Ecol.

[49] Baker MK, Ducasse FB (1967). Tick infestation of livestock in Natal. The predilection sites and seasonal variations of cattle ticks. J S Afr Vet Assoc.

[50] Walker AR, Bouattour A, Camicas JL, Estrada-Peña A, Horak IG, Latif A (2014). Ticks of domestic animals in Africa, A guide to identification of species.

[51] Guy EC, Stanek G (1991). Detection of *Borrelia burgdorferi* in patients with Lyme disease by the polymerase chain reaction. J Clin Pathol.

[52] Simpson V, Panciera R, Hargreaves J, McGarry J, Scholes S, Bown K (2005). Myocarditis and myositis due to infection with *Hepatozoon* species in pine martens (*Martes martes*) in Scotland. Vet Rec.

[53] Salih D, Hassan S, El Hussein A, Jongejan F (2004). Preliminary survey of ticks (Acari: Ixodidae) on cattle in northern Sudan. Onderstepoort J Vet Res.

[54] Silatsa B, Simo G, Githaka N, Mwaura S, Kamga R, Oumarou F (2019). A comprehensive survey of the prevalence and spatial distribution of ticks infesting cattle in different agro-ecological zones of Cameroon. Parasit Vect.

[55] Lawal MD, Ameh IG, Ahmed A (2007). Some ectoparasites of Camelus dromedarius in Sokoto Nigeria. Nig J Entomol.

[56] Onyiche TE, Răileanu C, Tauchmann O, Fischer S, Vasić A, Schäfer M (2020). Prevalence and molecular characterization of ticks and tick-borne pathogens of one-humped camels (*Camelus dromedarius*) in Nigeria. Parasit Vectors.

[57] Boka O, Achi L, Adakal H, Azokou A, Yao P, Yapi Y (2017). Review of cattle ticks (Acari, Ixodida) in Ivory Coast and geographic distribution of *Rhipicephalus (Boophilus) microplus*, an emerging tick in West Africa. Exp Appl Acarol.

[58] Nelson KS, Bwala DA, Nuhu EJ (2015). The dromedary camel; A review on the aspects of history, physical description, adaptations, behavior/lifecycle, diet, reproduction, uses. Genetics Dis Nig Vet J.

[59] Kabore H, Salembere M, Tamboura H (1998). Seasonal variation of ticks on cattle in Burkina Faso. Ann N Y Acad Sci.

[60] Sungirai M, Abatih E, Moyo D, Clercq P, Madder M (2016). Shifts in the distribution of ixodid ticks parasitizing cattle in Zimbabwe. Med Vet Entomol.

[61] Capek M, Literak I, Kocianova E, Sychra O, Najer T, Trnka A (2014). Ticks of the *Hyalomma marginatum* complex transported by migratory birds into Central Europe. Ticks Tick Borne Dis.

[62] Abdussamad AM, Holtz W, Gauly M, Suleiman MS, Bello MB. Reproduction and breeding in dromedary camels: Insights from pastoralists in some selected villages of the Nigeria-Niger corridor. Livestock Research for Rural Development. 2011;23. http://www.lrrd.org/lrrd23/8/abdu23178.htm. Accessed 20 Apr 2020.

[63] Oyewusi IK, Ganiyu IA, Akande FA, Takeet MI, Anifowoshe IO, Famuyide IM (2015). Assessment of ticks on cattle entering Nigeria through a major trans-boundary animal route in Ogun State. Bull Anim Health Prod Afr.

[64] Weir W, Ben-Miled L, Karagenç T, Katzer F, Darghouth M, Shiels B (2007). Genetic exchange and sub-structuring in *Theileria annulata* populations. Mol Biochem Parasitol.

[65] Gharbi M, Darghouth M, Elati K, Al-Hosary A, Ayadi O, Salih D (2020). Current status of tropical theileriosis in Northern Africa: A review of recent epidemiological investigations and implications for control. Transb Emerg Dis.

