# Correction to: First report of *Theileria annulata* in Nigeria: Findings from cattle ticks in Zamfara and Sokoto States

**DOI:** 10.1186/s13071-021-04818-y

**Published:** 2021-07-05

**Authors:** Adamu Haruna Mamman, Vincenzo Lorusso, Babagana Mohammed Adam, Goni Abraham Dogo, Kevin J. Bown, Richard J. Birtles

**Affiliations:** 1 School of Science, Engineering and Environment, The University of Salford, Greater Manchester, Salford, M5 4WT UK; 2 Department of Veterinary Parasitology and Entomology, Faculty of Veterinary Medicine, University of Jos, Plateau State, Jos, Nigeria; 3 Global Research & Intellectual Property Division, Vetoquinol, Paris, France

## Correction to: Parasites Vectors (2021) 14:242 https://doi.org/10.1186/s13071-021-04731-4

Following publication of the original article [1], it was brought to the authors’ attention that Goni Abraham Dogo had erroneously been affiliated with affiliation 3 instead of affiliation 2.

The original article has since been corrected and the updated author list can be found in this correction.

The authors apologize for any inconvenience caused.
